# Association of self-reported sleep duration with leukocyte telomere length in type 2 diabetes mellitus patients

**DOI:** 10.3389/fendo.2025.1549175

**Published:** 2025-05-22

**Authors:** Liqun Wang, Ruiping Pan, Ning Yan, Yiling Luo, Yali Wang

**Affiliations:** ^1^ Department of Epidemiology and Statistics, School of Public Health at Ningxia Medical University, Yinchuan, China; ^2^ Department of Chinese Medicine, The second people’s hospital of Shizuishan, Shizuishan, China; ^3^ Heart Center & Department of Cardiovascular Diseases, General Hospital of Ningxia Medical University, Yinchuan, China; ^4^ Department of Health Management Center, People’s Hospital of Ningxia Hui Autonomous Region, Yinchuan, China

**Keywords:** telomere length, sleep duration, sleep quality, type 2 diabetes mellitus patients, cross-sectional study

## Abstract

**Background:**

Leukocyte telomere length (LTL) is a biomarker of aging, and sleep duration is associated with LTL. However, research exploring the relationship between sleep duration and LTL has yielded inconsistent results. This study aimed to investigate the association between sleep duration and LTL in Chinese patients with type 2 diabetes mellitus (T2DM).

**Methods:**

A cross-sectional study involving 1,027 T2DM patients was conducted in Ningxia Province, China. Sleep duration was assessed through self-reported measures, while leukocyte telomere length (LTL) was determined using a quantitative polymerase chain reaction (q-PCR) method. Restricted cubic splines (RCS) analysis was initially performed to evaluate the potential nonlinear relationship between sleep duration and LTL. Subsequently, a multiple mixed-effect linear regression model was utilized to examine this association.

**Results:**

Binary analysis revealed an inverse association between sleep duration and LTL (β=-0.170, 95%*CI*: (-0.271, -0.068), *p*=0.001), indicating that for every 1-hour increase in sleep duration, LTL decreased by 0.17 kb. RCS analysis showed no evidence of a nonlinear relationship between sleep duration and LTL. After controlling for potential covariates, sleep duration remained negatively associated with LTL (β=-0.123, 95% *CI*: (-0.229, -0.017), *p*=0.022). When stratified by sleep quality (moderate or good vs. poor) and age (< 60 vs.≥60 years old), a negative association between sleep duration and LTL was particularly observed among individuals with moderate or good sleep quality and who were under 60 years old.

**Conclusion:**

This study adds to the growing literature relating sleep duration with biomarkers of aging and suggests that the shortening of LTL may reflect potential mechanisms through which longer sleep duration contributes to pathological conditions in T2DM patients. It is recommended that healthcare providers, health promoters, and patients pay greater attention to sleep habits, avoiding excessively long sleep durations, to potentially slow cellular aging.

## Introduction

Type 2 diabetes mellitus (T2DM) is widely recognized as an age-related condition influenced by a combination of genetic and environmental factors (1). Telomeres are DNA-protein complexes with repeat sequences of DNA, are special chromatin structures at the ends of eukaryotic chromosomes located at the termini of eukaryotic chromosomes, and protect the chromosome ends against fusion and degradation (2). Leukocyte telomere length (LTL) has been extensively studied as a biomarker of biological aging (3). On average, human telomeres shorten by 50 to 100 base pairs with each mitotic division (4). Epidemiological studies have demonstrated that shorter telomeres are linked to various age-related disorders, including coronary heart diseases (5), cognitive decline (6), and metabolic syndrome (7). Given that shorter LTL is driven by oxidative stress and inflammation (8), LTL is also linked to diabetes (9). In fact, the rate of telomere attrition is influenced by various environmental factors beyond mitotic replication rates (10). The influencing factors for LTL include controllable and uncontrollable factors. Among them, sleep conditions have garnered significant attention as a key modifiable factor.

Sleep is a vital psychological and biochemical process in humans, playing an essential role in numerous critical functions, including modulation of immune responses, disease, and psychological state (11). Abnormal sleep rhythm, whether characterized by shorter or longer sleep durations, as well as poor sleep quality, are associated with an increased susceptibility to declines in both physical and mental functioning (12, 13). These sleep disturbances are also predictive of age-related diseases, such as cardiovascular disease (14), diabetes (15), Alzheimer’s disease (16), early mortality (17), while additionally influencing molecular pathways related to inflammation and biological aging. In terms of LTL, a number of studies have reported a linear association between sleep duration and LTL, with shorter sleep duration linked to shorter telomere length among healthy participants (18, 19). However, inconsistent findings have also been reported, highlighting the need for a more comprehensive evaluation of the relationship between sleep duration and LTL. Some studies have found no significant association between sleep duration and telomere length (20), while recent research suggested that, compared to participants sleeping 7–8 hours, those sleeping longer than 8 hours had shorter LTL in middle-aged and young-old adults (21).

In summary, research findings on the association between sleep duration and telomere length remain inconsistent, with the majority of those studies focusing on healthy adults. Few studies have explored the relationship between sleep duration and LTL in T2DM patients. Hence, the present study was designed to evaluate the relationship between sleep duration and LTL using data from Chinese T2DM patients. This study is expected to provide an opportunity to enrich the research pool about sleep parameters and telomere length in T2DM patients.

## Materials and methods

### Subjects and procedure

**Figure 1:**
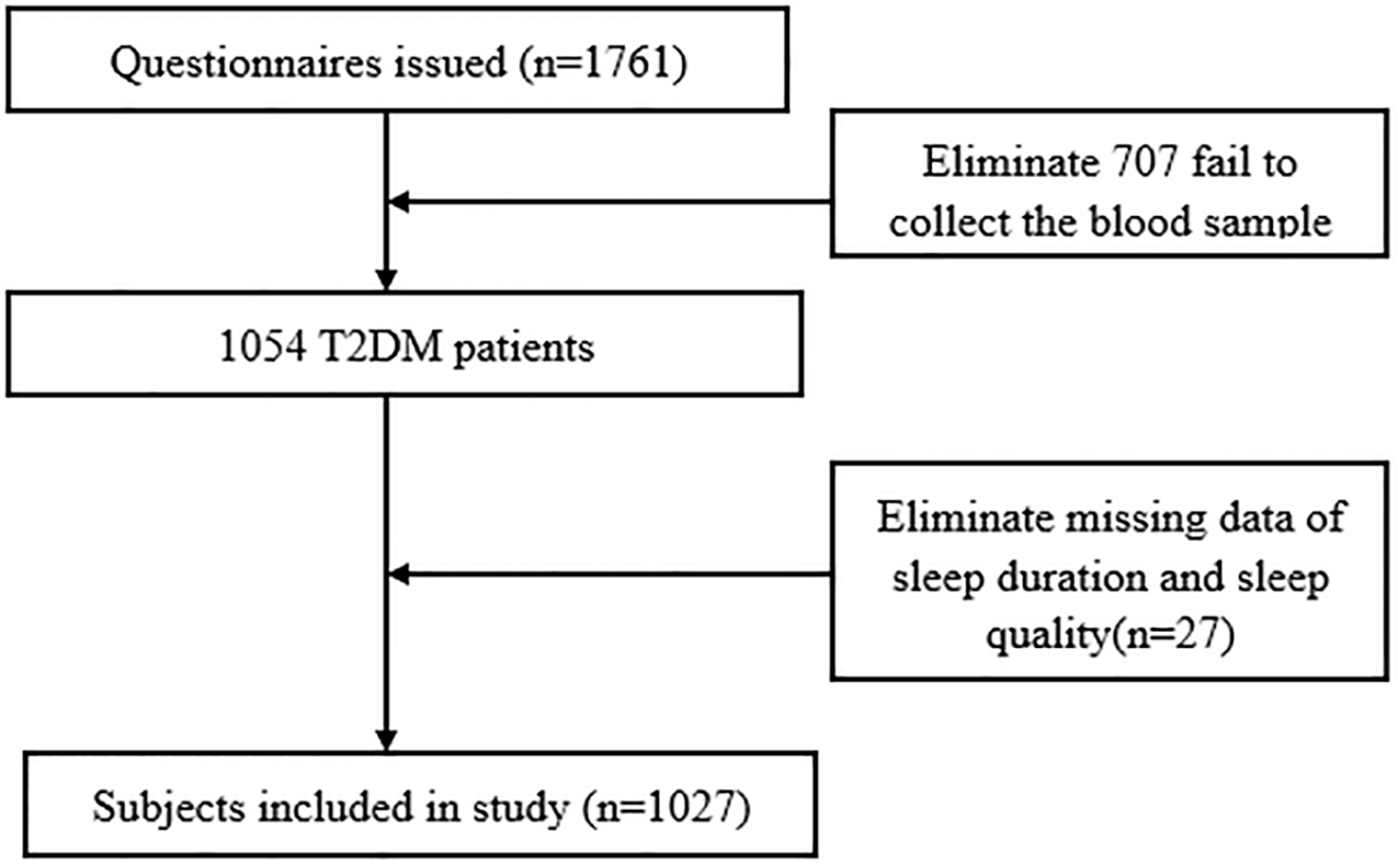
Flowchart for participants selection.

The study participants were recruited from a cross-sectional study conducted based on seven public hospitals in Ningxia province from August 2019 to November 2020. Detailed information regarding the study design and procedures is available in a previous study (22). Briefly, an eligible study sample was selected using a probability proportionate to size (PPS) sampling method. A total of 1,761 T2DM patients were initially included in this study. Of those, 707 patients were excluded due to the inability to collect blood samples, and an additional 27 patients were excluded because of missing information on sleep duration and sleep quality. Consequently, the final sample size for the present study consisted of 1,027 T2DM patients. A flowchart illustrating the data exclusion process is provided in **Figure 1**. The study was approved by the institutional review board of the Yinchuan Hospital of Traditional Chinese Medicine, along with the 1964 Helsinki Declaration and the later amendments or similar ethical standards. All participants provided written informed consent.

### Telomere length assay

The principle of the laboratory method has been previously described (23). In short, DNA was isolated from peripheral blood leukocytes, and the DNA concentration was quantified using a Nanodrop SD-1000 spectrophotometer. The DNA samples were spun down and resuspended to ensure accurate and uniform concentrations. The purified DNA samples with an OD260/OD280 ratio ranging from 1.7 to 2.1 were diluted with PCR-grade water (free of DNase and RNase) and transferred into 96-well plates at a fixed concentration of 5 ng/µl. Ratios within the specified range indicated low levels of contaminating protein in the nucleic acid. This assay involved comparing the copy number of the telomere repeat sequence (T) to that of a single-copy reference gene (36B4) (S) in each sample. The leukocyte telomere length (LTL) was determined using a quantitative polymerase chain reaction (qPCR) assay (24). The designs used the following primer sequences: 5’CGGTTTGTTTGGGTTTGGGTTTGGGTTTGG GTTTGGGTT3’ (forward), 5’GGCTTGCCTTACCCTTACCCTTACCCTTACCCTTACCCT 3’ (reverse) for telomere sequence amplification and 5’CAGCAAGTGGGA AGGTGTAATC C3’ (forward), 5’CCCATTCTATCATCAACGGGTACAA 3’ (reverse) for 36B4 sequence amplification.

Relative TL was calculated using the following formula: (TL=2^(△Ct2-△Ct1)^, where △Ct2 is the Ct(T)-Ct (36B4) of the sample and △Ct1 is the Ct(T)-Ct (36B4) of the standard. The Ct value is the point on the amplification curve where the fluorescent signal increases exponentially (25). The T/S ratio was calculated from the average quantity of reference DNA found to match with each experimental sample for the copy number of the targeted template (for T: the number of telomere repeats, and for S: the number of 36B4 gene copies) (26).

### Sleep duration

The main exposure variables were self-reported sleep duration, which was assessed by asking participants two questions, “What time do you usually go to sleep at night?” and “What time do you usually rise in the morning?”. Sleep duration was then calculated based on their responses. Participants who responded ‘don’t know’ or ‘refused’ were considered to have missing responses.

### Covariates

We selected covariates that could potentially influence the relationship between sleep duration and telomere length (27, 28). Those covariates included sociodemographic and health-related variables. Sociodemographic variables, known to be associated with both LTL and sleep duration, included age (continuous data), gender (male, female), education level (illiterate, primary school, junior school, college degree or above), marital status (unmarried, married, and widowed or divorced), Family per capita monthly income (FCMI, classified as <1,000 RMB, 1,000-1,999 RMB, 2,000-2,999 RMB, 3,000-4,999 RMB, and 5,000 RMB or more). Health-related variables, specifically, included smoking, defined as at least one cigarette per day and last for six months or more (yes vs. no), alcohol use, defined as a drink at least one glass of alcohol, that equal to 1/2 bottle of beer or 125-milliliter grape wine or fruit wine or 40-milliliter white wine, in the past 12 months (yes vs. no), sitting time (How much time do you usually spend sitting or reclining on a typical day except sleeping time)?, body mass index (calculated as body weight in kilograms divided by the square of height in meters), take a nap (yes vs. no), sleep quality (poor, moderate, good), T2DM complication (yes vs. no), take glucose lowering medications (yes vs. no), diabetes duration (continuous data), life satisfaction, defined as how satisfied are you with your current living situation (very satisfied, basically satisfied, fair, dissatisfied, very dissatisfied), eating sweet food (often, sometimes, rarely), consumption of oil (moderate, greasy, light), a balanced mix of meat and vegetables (mainly meat, mainly vegetarian, half meat and half vegetables). Serum triglycerides, total cholesterol, urea, and creatinine were tested in the hospital laboratory using standard procedures.

### Statistical analysis

The Kolmogorov-Smirnov test was used to assess the normality of continuous data. Normally distributed variables were expressed as mean (standard deviation), while non-normally distributed variables were expressed as median (interquartile range). Categorical variables were presented as numbers (percentage, %). Continuous variables with normal distribution were evaluated using Analysis of Variance, whereas those with non-normal distribution were tested using the Kruskal-Wallis test. Categorical variables were compared using the Chi-Squared test. To evaluate the potential nonlinear relationship between sleep duration and leukocyte telomere length (LTL), restricted cubic splines (RCS) analysis was performed. And a piecewise linear regression model was performed to calculate the threshold effect of sleep duration on LTL. Subsequently, multiple mixed-effect linear regression models were used to examine the association between sleep duration and LTL. Three separate models were constructed to adjust for covariates incrementally: Model 1 analyzed sleep duration independently; Model 2 adjusted for sociodemographic variables (age, gender, marital status, education, FCMI); and Model 3 built upon Model 2 by additionally adjusting for health-related variables (smoking, alcohol use, sitting time, body mass index, take a nap, sleep quality, T2DM complications, take glucose lowering medications, diabetes duration, life satisfaction, eat sweet food, consumption of oil, a balanced mix of meat and vegetables, triglycerides, total cholesterol, urea, and creatinine). The rural/urban was fitted as a random intercept model of the independent variables. Due to the possible interaction of sleep quality and sleep duration, we performed the regression process stratified by sleep quality. All the analyses were performed using the Software for Statistics and Data Science (STATA) 14.0.

## Results

### Sociodemographic characteristics of the participants

The characteristics of the study population according to LTL tertiles (T1: <0.58, T2: 0.58-2.00, T3: >2.00) were shown in the **Table 1**. Compared with the lowest group, participants in the highest group were more likely to be younger, live in urban areas, have high educational level, have high income level, smoking, drinking, take a nap, have good sleep quality and have less long sleep duration.

**Table 1:** The demographic characteristics of the participants.

Variables	Total(n=1027)	T1(n=342)	T2 (n=342)	T3 (n=343)	*p* value
Age, mean (SD), years	57.8 (12.0)	60.0 (12.0)	57.4 (11.6)	56.3 (12.0)	<0.001
Gender, male, n (%)	569 (55.4)	185 (54.1)	189 (55.3)	195 (56.9)	0.767
Marital status, married, n (%)	961 (93.6)	324 (94.7)	316 (92.4)	321 (93.6)	0.459
Education level, illiterate, n (%)	166 (16.2)	88 (25.7)	42 (12.3)	36 (10.5)	<0.001
FCMI, >5000, n (%)	126 (12.3)	28 (8.2)	50 (14.6)	48 (14.0)	<0.001
Rural, yes, n (%)	314 (30.6)	162 (47.4)	77 (22.5)	75 (21.9)	<0.001
Smoking, yes, n (%)	251 (24.4)	62 (18.1)	88 (25.7)	101 (29.4)	0.002
Alcohol use, yes, n (%)	256 (24.9)	66 (19.3)	92 (26.9)	98 (28.6)	0.011
Sitting time, mean (SD), hours	5.0 (2.5)	4.9 (2.8)	5.0 (2.3)	5.0 (2.4)	0.745
Body mass index, mean (SD), kg/m2	24.8 (3.5)	24.8 (3.5)	25.0 (3.5)	24.7 (3.5)	0.293
Take a nap, yes, n (%)	492 (47.9)	134 (39.2)	169 (49.4)	189 (55.1)	<0.001
Sleep quality, good, n (%)	342 (33.3)	111 (32.5)	108 (31.6)	123 (35.9)	0.005
T2DM complication, yes, n (%)	605 (58.9)	190 (55.6)	220 (64.3)	195 (56.9)	0.052
Take glucose lowering medications yes, n (%)	857 (83.4)	283 (82.7)	282 (82.5)	292 (85.1)	0.586
Diabetes duration, mean (SD), years	8.6 (7.6)	8.0 (7.4)	8.9 (7.9)	9.0 (7.4)	0.137
Life satisfaction, very satisfied, n (%)	92 (9.0)	35 (10.2)	29 (8.5)	28 (8.2)	0.952
Eating sweet food, often, n (%)	55 (5.4)	17 (5.0)	18 (5.3)	20 (5.8)	0.774
Consumption of oil, moderate, n (%)	193 (18.8)	64 (18.7)	64 (18.7)	65 (19.0)	0.025
A balanced mix of meat and vegetables, mainly meat, n (%)	137 (13.3)	40 (11.7)	52 (15.2)	45 (13.1)	0.419
Triglycerides, mean (SD), mmol/L	2.3 (2.1)	2.3 (1.9)	2.3 (2.4)	2.2 (1.9)	0.826
Total cholesterol, mean (SD), mmol/L	5.0 (15.4)	6.1 (26.6)	4.5 (1.2)	4.4 (1.1)	0.293
Urea, mean (SD), mmol/L	5.6 (2.0)	5.6 (1.9)	5.7 (2.3)	5.7 (1.9)	0.513
Creatinine, mean (SD), umol/L	67.2 (45.9)	68.1 (35.1)	67.0 (56.7)	66.5 (43.5)	0.906
Sleep duration, >9 hours, n (%)	121 (11.8)	55 (16.1)	34 (9.9)	32 (9.3)	0.022

T1: <0.58, T2:0.58-2.00, T3: >2.00; SD=Standard deviation, FCMI=Family per capita monthly income; T2DM= type 2 diabetes mellitus.

### Association between night sleep duration and LTL

Sleep duration was analyzed as a continuous variable using RCS (**Supplementary Figure S1**), which set up 7.5 h sleep duration as a reference and fitted with 4 knots (25th, 50th, 75th, and 95th percentiles). The results showed there was not the nonlinear relationship existed between sleep duration and LTL (P for nonlinear =0.226).

**Figure 2:**
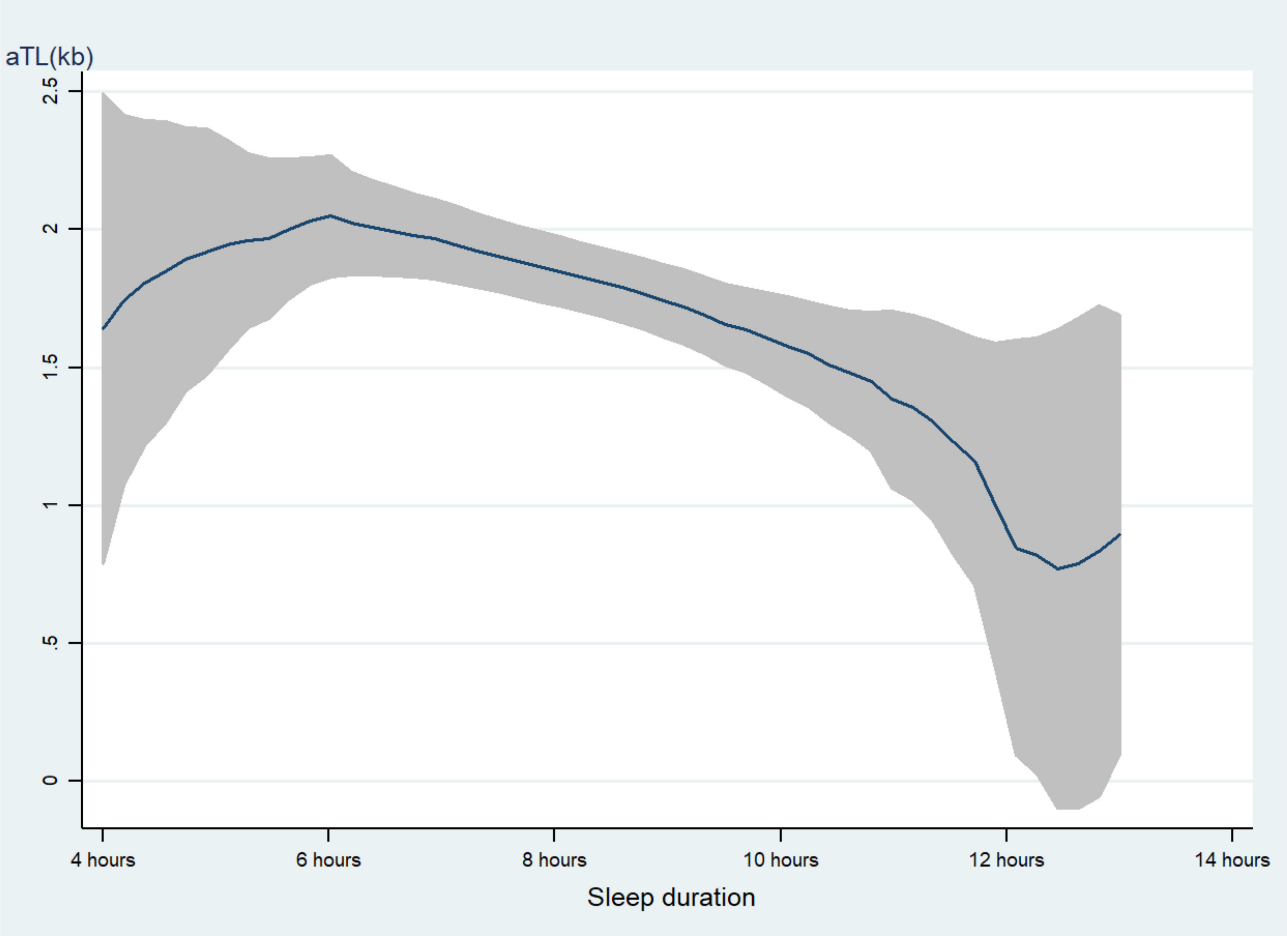
the level of LTL with the change of sleep duration. with the increasing of sleep duration, the LTL was shorter line represent the value of LTL; the gray confidence intervals represent the 95% confidence intervals of LTL.

As shown in **Table 2**, educational level, family income, smoking, alcohol use, and take a nap were positively associated with LTL. Age and sleep duration were negatively associated with LTL. Our piecewise linear model analysis showed no significant slope differences between sleep duration categories (<6h, 6-10h, and >10h) after covariates adjustment (**Supplementary Table S1**). As displayed in **Figure 2**, with too long (> 10 h) sleep duration was associated with shorter LTL.

**Table 2:** Bivariate regression model (n=1027).

Variables	β	SE	95%CI	P value
Age	-0.015	0.005	(-0.024, -0.005)	0.004
Gender (reference=female)	0.010	0.118	(-0.222, 0.242)	0.932
Marital status (reference=unmarried)	-0.191	0.239	(-0.661, 0.279)	0.427
Education level (reference=non-illiterate)	0.324	0.057	(0.211, 0.437)	<0.001
FCMI (reference=FCMI<1000)	0.096	0.047	(0.004, 0.188)	0.040
Smoking (reference=no)	0.441	0.136	(0.174, 0.708)	0.001
Alcohol use (reference=no)	0.314	0.136	(0.048, 0.580)	0.021
Sitting time (reference=no)	0.017	0.024	(-0.031, 0.064)	0.486
Body mass index	-0.005	0.016	(-0.038, 0.027)	0.744
Take a nap (reference=no)	0.469	0.116	(0.240, 0.699)	<0.001
Sleep quality (reference=poor)	-0.016	0.078	(-0.169, 0.137)	0.838
T2DM complication (reference=no)	-0.043	0.119	(-0.278, 0.191)	0.717
Take glucose lowering medications (reference=no)	0.138	0.158	(-0.172, 0.448)	0.383
Diabetes duration	0.009	0.007	(-0.006, 0.024)	0.255
Life satisfaction (reference=no)	0.075	0.100	(-0.122, 0.272)	0.455
Consumption of sweet food (reference=often)	-0.054	0.099	(-0.248,0.140)	0.584
Consumption of oil (reference=moderate)	-0.080	0.069	(-0.216, 0.056)	0.250
A balanced mix of meat and vegetables (reference=mainly meat)	0.083	0.083	(-0.079,0.245)	0.315
Triglycerides	-0.012	0.027	(-0.066,0.041)	0.650
Total cholesterol	-0.003	0.004	(-0.011,0.004)	0.394
Urea	0.001	0.029	(-0.055,0.058)	0.968
Creatinine	0.001	0.001	(-0.001,0.004)	0.353
Sleep duration	-0.170	0.052	(-0.271, -0.068)	0.001

FCMI, Family per capita monthly income; T2DM, type 2 diabetes mellitus; SE, Standard error; 95%CI= 95% confidence interval.

### Multiple mixed-effect linear regression model between sleep duration and LTL

As shown in **Table 3**, in model 1, there is an inverse association between sleep duration and LTL (β=-0.146, 95% *CI*: (-0.247, -0.045), *p*=0.004). The association persisted when controlling for sociodemographic variables (Model 2) (β=-0.110, 95% *CI*: (-0.212, -0.006), *p*=0.037), and health-related variables (Model 3) (β=-0.123, 95% *CI*: (-0.229, -0.017), *p*=0.022).

**Table 3:** Multiple mixed-effect linear regression model between sleep duration and leukocyte telomere length in T2DM patients (n=1027).

Variables	Model 1		Model 2		Model 3	
	β(95%CI)	P value	β(95%CI)	P value	β(95%CI)	P value
Sleep duration	-0.146(-0.247, -0.045)	0.004	-0.110(-0.212, -0.006)	0.037	-0.123(-0.229, -0.017)	0.022
Age	NA	NA	-0.008(-0.018, 0.003)	0.153	-0.012(-0.023, -0.001)	0.046
Gender (reference=female)	NA	NA	-0.283(-0.532, -0.034)	0.026	-0.526(-0.819, -0.234)	<0.001
Marital status (reference=unmarried)	NA	NA	-0.228(-0.692, 0.237)	0.337	-0.290(-0.789, 0.209)	0.255
Education level (reference=non-illiterate)	NA	NA	0.275(0.129, 0.420)	<0.001	0.258(0.110, 0.406)	0.001
FCMI (reference=FCMI<1000)	NA	NA	-0.032(-0.132, 0.067)	0.528	0.009(-0.097, 0.115)	0.869
Smoking (reference=no)	NA	NA	NA	NA	0.534(0.207,0.861)	0.001
Alcohol use (reference=no)	NA	NA	NA	NA	0.099(-0.219,0.419)	0.541
Sitting time (reference=no)	NA	NA	NA	NA	0.025(-0.023,0.073)	0.315
Body mass index	NA	NA	NA	NA	0.009(-0.154, 0.172)	0.911
Take a nap (reference=no)	NA	NA	NA	NA	0.393(0.152, 0.634)	0.001
Sleep quality (reference=poor)	NA	NA	NA	NA	0.005(-0.159, 0.169)	0.948
T2DM complication (reference=no)	NA	NA	NA	NA	-0.125(-0.380, 0.129)	0.333
Take glucose lowering medications (reference=no)	NA	NA	NA	NA	0.209(-0.108, 0.527)	0.196
Diabetes duration	NA	NA	NA	NA	0.012(-0.006, 0.030)	0.171
Life satisfaction (reference=no)	NA	NA	NA	NA	0.150(-0.056,0.356)	0.153
Consumption of sweet food (reference=often)	NA	NA	NA	NA	0.002(-0.197,0.201)	0.985
Consumption of oil (reference=moderate)	NA	NA	NA	NA	0.027(-0.114,0.169)	0.704
A balanced mix of meat and vegetables (reference=mainly meat)	NA	NA	NA	NA	0.175(0.008,0.342)	0.039
Triglycerides	NA	NA	NA	NA	-0.033(-0.087,0.021)	0.232
Total cholesterol	NA	NA	NA	NA	-0.002(-0.001,0.004)	0.657
Urea	NA	NA	NA	NA	-0.020(-0.085,0.045)	0.547
Creatinine	NA	NA	NA	NA	0.001(-0.001,0.004)	0.269

FCMI=Family per capita monthly income; T2DM= type 2 diabetes mellitus; Model l =sleep duration separately; Model 2 = Model l + sociodemographic variables (age, gender, marital status, education, FCMI); Model 3 = Model 2 + health-related variables (smoking, alcohol use, sitting time, body mass index, take a nap, sleep quality, T2DM complications, take glucose lowering medications, diabetes duration, life satisfaction, eat sweet food, consumption of oil, a balanced mix of meat and vegetables, triglycerides, total cholesterol, urea, and creatinine); 95%CI= 95% confidence interval; NA, not apply.

### Multiple mixed-effect linear regression model between sleep duration and LTL stratified by sleep quality

As shown in **Table 4**, when stratified by sleep quality (poor vs. moderate or good). A negative association between sleep duration and LTL (β=-0.134, 95% *CI*: (-0.260, -0.009), *p* =0.036) was found among participants with moderate or good sleep quality.

**Table 4:** Multiple mixed-effect linear regression model between sleep duration and leukocyte telomere length in T2DM patients stratified by sleep quality.

Variables	Poor sleep quality (n=246)		Moderate or good sleep quality (n=781)	
	β (95%CI)	P value	β (95%CI)	P value
Sleep duration	-0.063 (-0.257, 0.132)	0.529	-0.134 (-0.260, -0.009)	0.036
Age	-0.020 (-0.047, 0.007)	0.154	-0.012 (-0.025, 0.001)	0.075
Gender (reference=female)	-0.826 (-1.501, -0.151)	0.016	-0.353 (-0.679, -0.027)	0.034
Marital status (reference=unmarried)	-0.582 (-1.422, 0.258)	0.174	-0.073 (-0.711, 0.563)	0.821
Education level (reference=non-illiterate)	0.227 (-0.086, 0.540)	0.155	0.266 (0.103, 0.428)	0.001
FCMI (reference=FCMI<1000)	0.033 (-0.192, 0.259)	0.771	0.026 (-0.092, 0.145)	0.661
Smoking (reference=no)	-0.078 (-0.845, 0.688)	0.841	0.645 (0.286, 1.005)	<0.001
Alcohol use (reference=no)	0.306 (-0.444, 1.057)	0.423	0.087 (-0.265, 0.438)	0.629
Sitting time (reference=no)	-0.010 (-0.113, 0.092)	0.846	0.047 (-0.007, 0.101)	0.092
Body mass index	0.004 (-0.331, 0.339)	0.981	-0.023 (-0.207, 0.162)	0.808
Take a nap (reference=no)	0.095 (-0.428, 0.618)	0.721	0.512 (0.242, 0.779)	<0.001
T2DM complication (reference=no)	-0.479 (-1.028, 0.070)	0.087	-0.108 (-0.394, 0.177)	0.456
Take glucose lowering medications (reference=no)	0.019 (-0.688, 0.726)	0.958	0.233 (-0.120, 0.587)	0.196
Diabetes duration	0.010 (-0.023, 0.044)	0.530	0.019 (-0.002, 0.040)	0.070
Life satisfaction (reference=no)	-0.029 (-0.405, 0.347)	0.880	0.224 (-0.015, 0.464)	0.066
Consumption of sweet food (reference=often)	0.070 (-0.373,0.514)	0.756	-0.034 (-0.256,0.187)	0.761
Consumption of oil (reference=moderate)	0.071 (-0.218,0.360)	0.630	0.013 (-0.151,0.176)	0.879
A balanced mix of meat and vegetables (reference=mainly meat)	0.202 (-0.176,0.580)	0.295	0.166 (-0.017,0.350)	0.076
Triglycerides	-0.086 (-0.269,0.096)	0.355	-0.030 (-0.086,0.026)	0.299
Total cholesterol	0.069 (-0.163,0.302)	0.559	-0.002 (-0.009,0.005)	0.560
Urea	-0.149 (-0.270,-0.027)	0.016	-0.036 (-0.042,0.114)	0.367
Creatinine	0.009 (0.004,0.015)	0.001	-0.001 (-0.004,0.002)	0.610
Sleep duration	-0.063 (-0.257, 0.132)	0.529	-0.134 (-0.260, -0.009)	0.036
Age	-0.020 (-0.047, 0.007)	0.154	-0.012 (-0.025, 0.001)	0.075
Gender (reference=female)	-0.826 (-1.501, -0.151)	0.016	-0.353 (-0.679, -0.027)	0.034
Marital status (reference=unmarried)	-0.582 (-1.422, 0.258)	0.174	-0.073 (-0.711, 0.563)	0.821
Education level (reference=non-illiterate)	0.227 (-0.086, 0.540)	0.155	0.266 (0.103, 0.428)	0.001
FCMI (reference=FCMI<1000)	0.033 (-0.192, 0.259)	0.771	0.026 (-0.092, 0.145)	0.661
Smoking (reference=no)	-0.078 (-0.845, 0.688)	0.841	0.645 (0.286, 1.005)	<0.001
Alcohol use (reference=no)	0.306 (-0.444, 1.057)	0.423	0.087 (-0.265, 0.438)	0.629
Sitting time (reference=no)	-0.010 (-0.113, 0.092)	0.846	0.047 (-0.007, 0.101)	0.092
Body mass index	0.004 (-0.331, 0.339)	0.981	-0.023 (-0.207, 0.162)	0.808
Take a nap (reference=no)	0.095 (-0.428, 0.618)	0.721	0.512 (0.242, 0.779)	<0.001
T2DM complication (reference=no)	-0.479 (-1.028, 0.070)	0.087	-0.108 (-0.394, 0.177)	0.456
Take glucose lowering medications (reference=no)	0.019 (-0.688, 0.726)	0.958	0.233 (-0.120, 0.587)	0.196
Diabetes duration	0.010 (-0.023, 0.044)	0.530	0.019 (-0.002, 0.040)	0.070
Life satisfaction (reference=no)	-0.029 (-0.405, 0.347)	0.880	0.224 (-0.015, 0.464)	0.066
Consumption of sweet food (reference=often)	0.070 (-0.373,0.514)	0.756	-0.034 (-0.256,0.187)	0.761
Consumption of oil (reference=moderate)	0.071 (-0.218,0.360)	0.630	0.013 (-0.151,0.176)	0.879
A balanced mix of meat and vegetables (reference=mainly meat)	0.202 (-0.176,0.580)	0.295	0.166 (-0.017,0.350)	0.076
Triglycerides	-0.086 (-0.269,0.096)	0.355	-0.030 (-0.086,0.026)	0.299
Total cholesterol	0.069 (-0.163,0.302)	0.559	-0.002 (-0.009,0.005)	0.560
Urea	-0.149 (-0.270,-0.027)	0.016	-0.036 (-0.042,0.114)	0.367
Creatinine	0.009 (0.004,0.015)	0.001	-0.001 (-0.004,0.002)	0.610

FCMI, Family per capita monthly income; T2DM, type 2 diabetes mellitus.

### Multiple mixed-effect linear regression model between sleep duration and LTL stratified by age

As shown in **Table 5**, when stratified by age, a negative association between sleep duration and LTL (β=-0.145, 95% *CI*: (-0.287, -0.002), *p* =0.047) was found among those who under 60 years old.

**Table 5:** Multiple mixed-effect linear regression model between sleep duration and leukocyte telomere length in T2DM patients stratified by age.

Variables	Age<60 years old (n=567)		Age≥60 years old (n=460)	
	β (95%CI)	P value	β (95%CI)	P value
Sleep duration	-0.145 (-0.287, -0.002)	0.047	-0.119 (-0.274, 0.035)	0.129
Gender (reference=female)	-0.528 (-0.939, -0.117)	0.012	-0.634 (-1.061, -0.208)	0.004
Marital status (reference=unmarried)	0.094 (-0.752, 0.941)	0.827	-0.525 (-0.156, 0.104)	0.102
Education level (reference=non-illiterate)	0.125 (-0.079, 0.330)	0.229	0.431 (0.226, 0.635)	<0.001
FCMI (reference=FCMI<1000)	0.047 (-0.095, 0.189)	0.518	-0.038 (-0.197, 0.120)	0.637
Smoking (reference=no)	0.339 (0.060, 0.737)	0.096	1.026 (0.455, 1.596)	<0.001
Alcohol use (reference=no)	0.265 (-0.127, 0.656)	0.185	-0.128 (-0.684, 0.428)	0.651
Sitting time (reference=no)	0.059 (-0.008, 0.126)	0.087	-0.007 (-0.076, 0.062)	0.845
Body mass index	-0.002 (-0.220, 0.216)	0.986	0.052 (-0.196, 0.302)	0.677
Take a nap (reference=no)	0.403 (0.084, 0.722)	0.013	0.346 (0.020, 0.713)	0.064
T2DM complication (reference=no)	-0.405 (-0.957, 0.147)	0.150	0.159 (-0.240, 0.558)	0.434
Sleep quality (reference=poor)	-0.096 (0.318,0.127)	0.401	0.096 (-0.146,0.339)	0.436
Take glucose lowering medications (reference=no)	0.067 (-0.352, 0.486)	0.753	0.287 (-0.203, 0.777)	0.251
Diabetes duration	0.007 (-0.025, 0.041)	0.654	-0.005 (-0.028, 0.017)	0.638
Life satisfaction (reference=no)	-0.070 (-0.449, 0.309)	0.718	0.098 (-0.211, 0.409)	0.533
Consumption of sweet food (reference=often)	0.026 (-0.231,0.283)	0.843	-0.049 (-0.361,0.262)	0.756
Consumption of oil (reference=moderate)	0.107 (-0.094,0.308)	0.297	-0.083 (-0.285,0.118)	0.417
A balanced mix of meat and vegetables (reference=mainly meat)	0.266 (0.061,0.470)	0.011	-0.015 (-0.304,0.274)	0.919
Triglycerides	0.026 (-0.231,0.283)	0.712	-0.079 (-0.212,0.053)	0.242
Total cholesterol	0.013 (-0.055,0.080)	0.073	-0.001 (-0.007,0.007)	0.944
Urea	0.026 (-0.075,0.126)	0.617	-0.047 (-0.134,0.040)	0.287
Creatinine	-0.001 (-0.003,0.003)	0.971	0.004 (-0.001,0.008)	0.090

FCMI, Family per capita monthly income; T2DM, type 2 diabetes mellitus.

## Discussion

This study examined associations of sleep duration with LTL in Chinese T2DM patients using data from seven hospitals. The results revealed that sleep duration was inversely associated with LTL among T2DM patients after controlling for potential confounders using a mixed effect linear regression model. The findings indicated that individuals with either shorter or longer sleep duration had shorter LTL. As shown in **Figure 2**, when the sleep duration exceeded 6 hours, there was a slight decrease, while when it exceeded 10 hours, the downward trend became more pronounced. This contrasts with previous research on the association between sleep duration and telomere length, where shorter sleep duration was associated with shorter LTL (10, 28). Nevertheless, other cross-sectional studies reported that longer sleep duration (>8 hours) was associated with shorter LTL (6, 29), which was consistent with our findings. Even the previous study found a U-shaped association between sleep duration and LTL, meaning that both shorter and longer sleep durations are significantly related to shorter telomere length (30). However, the potential biological mechanism by which long sleep duration shortens LTL is currently unclear. One possible explanation is that excessive sleep duration is associated with elevated levels of inflammatory cytokines, including interleukin-6 (IL-6), C-reactive protein (CRP), fibrinogen, etc. (31, 32). Inflammatory factors, such as IL-6 and TNF-α, are linked to shorter LTL (33). Some health risk factors may lead to prolonged sleep time and shortened LTL, individuals with longer sleep duration have been shown to present 50% to 80% higher relative mortality risk and illness risk (34, 35), especially our study sample were T2DM patients. Meanwhile, Short and long sleep durations were associated with an increased blood glucose level and hemoglobin A1c (36), altered glucose metabolism and further disturb the LTL. Furthermore, as sleep duration increases, more adverse events may emerge, such as elevated sleep fragmentation, compromised sleep perception, altered cytokine levels, and reduced exposure to mild stressors, which may accelerate the dissolution of telomeres. Additionally, in this study, the mean age of T2DM patients was 57.8 years old, the National Sleep Foundation recommends 7–8 h for older adults (37), even the American Academy of Sleep Medicine and Sleep Research Society recommends ≥7 h per night on a regular basis for adults aged 18–60 years (38). Therefore, this further clarifies that either insufficient or excessive sleep duration is not conducive to maintaining telomere length.

The stratified multivariate regression model in the present study also indicated an association between sleep duration and LTL, which was observed among individuals with moderate or good sleep quality. No significant association was found between sleep duration and LTL among individuals with poor sleep quality. Theoretically, good sleep quality and enough sleep duration are adequate for main health or telomere length. However, even with good sleep quality, prolonged sleep duration can still be detrimental to telomere maintenance. Meanwhile, the association between sleep duration and LTL is more significant in T2DM patients under 60 years old. The possible mechanism may be that individuals under the age of 60 are typically in the middle age stage, during which physiological changes may have a more sensitive impact on telomere length. As age increases, the natural shortening of telomeres may mask the impact of sleep duration on LTL. As well, those individuals may be more likely to maintain irregular lifestyle habits, which may interact with sleep duration and further affect LTL. Furthermore, our results displayed that educational level, family income, and taking a nap were positively associated with LTL, the results were consistent with other studies (39–41). Despite previous evidence suggesting that smoking and alcohol consumption were negatively associated with LTL (20), our study found a positive correlation between smoking, alcohol use and LTL. One possible reason for this discrepancy may be differences in study samples or methodologies. Regarding age, in line with other results (42), we also found that older age was significantly associated with shorter LTL.

The most prominent advantage of our study is its use of a patient-based, multi-stage random sampling design, along with a large sample size with LTL results. However, there still exist several limitations. Firstly, because the cross-sectional design does not allow causality, we can only infer association between sleep duration and telomere length. Secondly, sleep duration was collected via a self-reported item. It may involve information bias despite being commonly used in epidemiological studies due to feasibility considerations. Thirdly, our study population is Chinese T2DM patients, and due to the lack of inclusion of healthy individuals or other non-T2DM individuals, we cannot determine whether the negative correlation between LTL and self-reported sleep duration is only applicable to T2DM patients or equally applicable to the general healthy population. Thus, the results are bound to be carefully extrapolated to other populations. Future studies should include a non-T2DM control group and compare the differences between T2DM patients and healthy individuals.

## Conclusions

Longer sleep durations were associated with shorter LTL in T2DM patients, potentially reflecting accelerated biological aging. Moreover, these associations were stronger in participants with moderate or good sleep quality and in those under 60 years old. Hence, it is recommended that healthcare providers, health promoters, and patients pay closer attention to sleep habits, avoiding excessively long sleep durations, to potentially slow cellular aging.

## Data Availability

The raw data supporting the conclusions of this article will be made available by the authors, without undue reservation.

## Supplementary material

Supplementary Table 1 Piecewise linear regression analysis of sleep duration and LTL. SD: sleep duration. After control for age, gender, marital status, education, FCMI, smoking, alcohol use, sitting time, BMI, take a nap, sleep quality, T2DM complications, take glucose lowering medications, diabetes duration, life satisfaction, eat sweet food, consumption of oil, a balanced mix of meat and vegetables, triglycerides, total cholesterol, urea, and creatinine.
