# Supplementary figures and images for: Association of self-reported sleep duration with leukocyte telomere length in type 2 diabetes mellitus patients
Source: Front Endocrinol (Lausanne). 2025 May 22;16:1549175. doi: 10.3389/fendo.2025.1549175 (PMC12137104; doi:10.3389/fendo.2025.1549175)

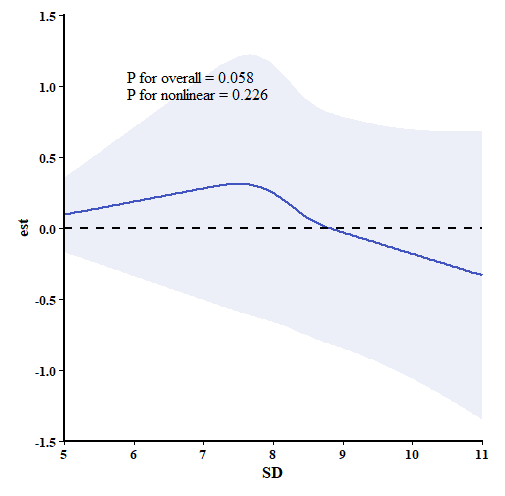

Supplement: Supplementary file 1 [file Image1.tiff]
