# Supplementary material for: Association of self-reported sleep duration with leukocyte telomere length in type 2 diabetes mellitus patients
Source: Front Endocrinol (Lausanne). 2025 May 22;16:1549175. doi: 10.3389/fendo.2025.1549175 (PMC12137104; doi:10.3389/fendo.2025.1549175)
Supplement: Supplementary Table 1 — Piecewise linear regression analysis of sleep duration and LTL. SD: sleep duration. After control for age, gender, marital status, education, FCMI, smoking, alcohol use, sitting time, BMI, take a nap, sleep quality, T2DM complications, take glucose lowering medications, diabetes duration, life satisfaction, eat sweet food, consumption of oil, a balanced mix of meat and vegetables, triglycerides, total cholesterol, urea, and creatinine. [file Table1.docx]

Table S1 Piecewise linear regression analysis of sleep duration and LTL

|  | β | SE | t | ***P*** value |
| --- | --- | --- | --- | --- |
| SD <6h | 0.377 | 0.389 | 0.970 | 0.332 |
| SD=6-10h | -0.115 | 0.065 | -1.775 | 0.076 |
| SD >10h | -0.574 | 0.307 | -1.869 | 0.062 |

SD: sleep duration. After control for age, gender, marital status, education, FCMI, smoking, alcohol use, sitting time, BMI, take a nap, sleep quality, T2DM complications, take glucose lowering medications, diabetes duration, life satisfaction, eat sweet food, consumption of oil, a balanced mix of meat and vegetables, triglycerides, total cholesterol, urea, and creatinine
